# The Melbourne Hospital

**Published:** 1904-09-24

**Authors:** 


### THE MELBOURNE HOSPITAL.

Considerable interest in hospital affairs has recently been aroused in Melbourne, brought about by the election of the committee of the Melbourne Hospital. It appears that a feeling had been growing that greater activity was required in the conduct of the affairs of the hospital. Although in low water as to funds, some of these complaints are unfair. Recently the committee showed its accessibility to new and improved modes of work by abandoning the obsolete and cumbrous method of keeping its accounts, practised almost since the foundation of the hospital in 1848, in favour of Sir Henry Burdett's "Uniform System of Accounts." The propriety of having a medical man on the committee of management is the latest improvement to which the hospital authorities approved. There is, however, a medical superintendent, who gives a weekly report to the committee, who are supposed to consult him on all subjects relating to his work. But this is said to be only a supposition, and the general dissatisfaction became apparent when the time approached for six members of committee to retire, though eligible for re-election for three years. Efforts were made to secure the election of fresh blood. The new candidates were Dr. William Moore, Melbourne's premier surgeon; Mr. George Gibb, president of the Caledonian Society, and one of the most successful and most highly esteemed business men of the city; and Mr. J. D. Carruthers, also engaged in commerce. The five or six thousand governors of the hospital responded by placing Mr. Gibb at the top of the poll and Dr. Moore second, and re-electing four of the old members. It was noticeable that the Chinese subscribers—of whom there are about 50, contributing one guinea and two guineas a year—were particularly assiduous and interested voters. Many governors and subscribers object to the present mode of electing the committee, and regard the distribution of votes as unfair.

